# Chemical Sensing Employing Plant Electrical Signal Response-Classification of Stimuli Using Curve Fitting Coefficients as Features

**DOI:** 10.3390/bios8030083

**Published:** 2018-09-10

**Authors:** Shre Kumar Chatterjee, Obaid Malik, Siddharth Gupta

**Affiliations:** 1 School of Electronics and Computer Science, University of Southampton, Southampton SO17 1BJ, UK; om1v10@soton.ac.uk; 2 Seamax Engineering Pte Ltd., Bengaluru 560017, India; siddugupta@gmail.com

**Keywords:** plant electrical signals, classification, curve fitting

## Abstract

In order to exploit plants as environmental biosensors, previous researches have been focused on the electrical signal response of the plants to different environmental stimuli. One of the important outcomes of those researches has been the extraction of meaningful features from the electrical signals and the use of such features for the classification of the stimuli which affected the plants. The classification results are dependent on the classifier algorithm used, features extracted and the quality of data. This paper presents an innovative way of extracting features from raw plant electrical signal response to classify the external stimuli which caused the plant to produce such a signal. A curve fitting approach in extracting features from the raw signal for classification of the applied stimuli has been adopted in this work, thereby evaluating whether the shape of the raw signal is dependent on the stimuli applied. Four types of curve fitting models—Polynomial, Gaussian, Fourier and Exponential, have been explored. The fitting accuracy (i.e., fitting of curve to the actual raw signal) depicted through R-squared values has allowed exploration of which curve fitting model performs best. The coefficients of the curve fit models were then used as features. Thereafter, using simple classification algorithms such as Linear Discriminant Analysis (LDA), Quadratic Discriminant Analysis (QDA) etc. within the curve fit coefficient space, we have verified that within the available data, above 90% classification accuracy can be achieved. The successful hypothesis taken in this work will allow further research in implementing plants as environmental biosensors.

## 1. Introduction

Our surrounding natural environment is one of the most important aspect in our lives, wherever we are geographically located in the world. The most basic and simple reason behind this being the supply of food, water and oxygen, which are required for our sustenance. Two of the main constituents of our surrounding environment are the flora—plants and trees which provide us with oxygen and fruits, the vital components required for us human beings, and fauna—the animals. Plants, trees and forests also provide us with cleaner air by removing a few harmful components from the atmosphere such as Ozone (O_3_), Sulphur dioxide (SO_2_) and Nitrogen dioxide (NO_2_) which may cause adverse neurological, cardio-vascular and pulmonary health effects in human beings [1]. 

As a precautionary measure, many institutions across the globe constantly monitor our environment for pollutants which may harm us and the flora/fauna, directly or indirectly. Such a monitoring system may present us with valuable real time information which can be used to control or even prevent any short/long term damage to the environment we are surrounded by and thereby improve our healthy sustenance. The monitoring systems, employed by various institutions, are usually used for sensing one or multiple environmental parameters and may cover a certain geographical area of interest. However, such monitoring over a large geographical area could be quite expensive and complex infrastructure oriented [2,3,4]. Therefore, not many countries may be enthusiastic to pursue it as much as others, thereby increasing the chances of long-term damage to their local as well as the overall global environment. In such a scenario, if a monitoring system is developed which is cost effective and can be implemented on a large scale then it becomes meritorious and worthy of being implemented by every nation for a justified cause of detecting harmful environmental pollutants which affect our quality of life. Such a holistic system seems like a possibility when we think about plant electrophysiology i.e., electrical signals generated by the plants in response to external stimuli. 

Plants possess sensing mechanisms which are employed to monitor the environment for optimal growth. This sensing mechanism can be observed by the change in behavior in plants like *Mimosa pudica* (touch me not) which closes its leaves when touched or *Dionaea muscipula* (Venus flytrap) which closes its trap when an insect gets in it. It has been established that plants produce an electrical signal response to stimuli which is used to control various physiological phenomena within the plant. These electrical signals, which may have embedded signatures capturing the essence of the stimuli affecting them, may be used as means of sensing the environment in which they grow. 

Excitability in plants occurs due to the high sensitivity of protoplasm and all cell organelles to any natural and electrochemical effects [5]. It is reported in Reference [6] that due to any type of stimulus, an initial influx of Ca^2+^ triggers a Cl^−^ efflux via anion channels which lead to massive and quick plasma membrane depolarization. A slow repolarization of the plasma membrane takes place due to activation of K^+^ efflux up to the resting potential. Due to ion channel gating, this electrical wave (due to influx and efflux of ions) propagates through the sieve tubes. 

Apart from a local electrical potential (LEP), which is induced and sustained due to a wounding/injury and stops a few millimeters from a dying cell, there are action potential (AP) and variation potential (VP) [7,8]. All these three types of signals are due to a transient change in the membrane potential of plant cells (depolarization/repolarization phases), but only VP’s and AP’s make use of the vascular bundles to systemically spread through the entire plant body [7,8]. Out of these three types of electrical signals, AP and VP are of particular interest and several works have been reported in analyzing them [9,10,11,12,13,14,15,16,17], including mathematical modelling of such signals [18]. When observing the trends in the plant electrical signal responses used in this exploration, some of the signal response were found to have a trend opposite to the direction of other plants for the same stimuli. One explanation for this could be system potential (SP) [19], which is a novel type of electrical signal which propagates systematically from leaf to leaf. These types of signals are different to AP/VP and depend upon the nature and intensity of the stimuli applied. It is also found that unlike the primary polarity of AP/VP, the polarity of SP is reversed [19].

Plants, covering around one third of earth’s land mass [20] in the form of forests, are found in abundance, thereby covering a large geographical area naturally. Plants are also affected by the same parameters (environmental pollutants) as other living beings sharing the same natural environment. Therefore, if electrical signals such as AP/VP/SP from each plant could be used to monitor a certain area around that particular plant, then their abundantly distributed presence could be used to monitor a larger area at the same time. The viability of such an approach seems immense, but several questions rise along with this. If such electrical signals are extracted and analyzed, information about the external stimuli which caused the electrical signal may be found. A successful analysis of such signals will assist the idea of plants being used as a living, multiple stimuli biosensor.

Classification of stimulus using raw plant electrical signals were carried out previously using various discriminant classification algorithms and statistical feature combinations [21], reporting a classification result of around 70%. These results were based on average binary classification results obtained using three different types of stimuli—Sulphuric acid (H_2_SO_4_), Sodium Chloride (NaCl) solution and Ozone (O_3_) in a laboratory environment. Similarly, a decision tree-based multiclass classification was explored in Reference [22]. However, in these two papers various statistical features have been extracted from small windows of the time series (plant electrical signals). The classification results reported in Reference [22] are above 90%, when using these statistical features computed from the small segments of the time series. The questions which arise at this point are:

- Are there any other methods of capturing the information about the applied stimuli from the plant electrical signal response? Will a new method produce similar or better classification accuracy than previously reported in Reference [22]?
- Can the entire duration of the time series be considered for providing more information about the applied stimuli? In other words, is there more information embedded within the entire duration of the time series data which is recorded, which aids in similar or better results than reported in previous studies?

To answer both these questions, we hypothesized that the shape of the raw plant electrical signal, as a response to an applied stimulus, will be different for different stimuli. Hence the feature extraction could be based on the shape of this raw signal. One way of carrying this out is through fitting a curve to the signal and treating the curve fitting coefficients as features. If successful, it will also validate that the shape of the electrical response of a plant is varied for different stimuli. As a method, a similar approach was taken in Reference [23] where the authors demonstrated usage of Mahalanobis distance classifier for classification of object trajectory-based video motion clips, assuming that the clusters of the trajectory points are distributed normally in the coefficient feature space.

The main contributions of this paper are:

- The identification of the hypothesis that the shape of the available plant electrical signals, acquired under laboratory conditions, are different for different stimuli is established.
- The definition of the features that can be used for classification through a curve fitting approach, where the coefficients of the curve fits were provided to simple classifier algorithms such as LDA, QDA etc.
- The validation of such curve fit coefficients as features that allow the achievement of classification results above 90%.

Thus, in this paper, we aim to explore a different feature set than what has been reported so far [21,22], so that optimal information for classification of the stimulus is obtained. This will also encourage the wider research community to take a step closer in realizing a holistic plant electrophysiology-based environmental monitoring system, which may help us take preventive measures against any harm to us or our natural environment, attributed to certain environmental pollutants, in a timely fashion. The majority of the work presented here is based on the chapter in the PhD thesis by the first author which can be accessed via Reference [24].

## 2. Stimuli Targeted for This Exploration

(a) One of the major components of air pollution today is tropospheric/ground level Ozone (O_3_) [25], which is the product of volatile organic compounds (VOCs) and Nitrogen oxides (NOx) aided by a rise in ambient temperature. Increasing ground level O_3_, which is approximately 1.66 times heavier than air [26], is causing serious damage to crops, forest cover and human health and has been a topic of research in different parts of the world [25]. The tropospheric O_3_ is thus a secondary pollutant, which can be contributed to by multiple sources. It is also suggested that precursors of O_3_ found in the USA were emitted in Asia [27], thereby suggesting that the pollutant may not be necessarily locally contributed. Monitoring of tropospheric O_3_ has been picked up in different parts of the world for its perceived adverse health impact [25,26,27,28,29,30,31,32]. Previous research on the detection of Ozone level through plant activity [33] and recent advances in realization of plant electrophysiology-based biosensors through a distributed network of wireless devices connected to the plants [34] highlights the importance of focusing on Ozone as an environmental pollutant.
(b) Similarly, salinity of the soil is a major concern, especially for agriculture as it leads to reduction in crop yield [35]. Apart from affecting crops/vegetation on an immediate basis, there is a long term effect of degradation of the soil which is considered as irreversible [35]. The amount of croplands, estimated to be around one third of the total current area, is expected to increase with a rise in global climate change [35]. One of the major components of salinity in the soil is common salt or sodium chloride (NaCl). When NaCl permeates the soil due to some reason, it can be harmful to both plants and organisms such as snails, slugs, frogs, newts and earthworms thriving in the soil [36]. It can also alter the salinity of the groundwater table underneath and will thus increase the salinity of drinking water which will harm human and animal health too [37]. If the salt reaches nearby fresh water streams, ponds, lakes etc. It will not only affect the aquatic life [36] living in such waterbodies but also affect the animals and human beings exposed to such water resources. Apart from being a health hazard and being damaging to the ecosystem, increased NaCl concentrations can also be corrosive to vehicles and infrastructure such as bridges etc. There could be several sources of NaCl input to the soil including usage of water softeners, septic/sewage effluent and de-icing of roads/highways in winter months, etc. [37]. The amount and scope of usage of common salt as de-icer for roads during winter months in locations across the globe makes it a major contributor for soil pollution [6,37,38,39,40,41,42,43,44,45,46].
(c) The admixture of wet and dry deposited material from the atmosphere with abnormal amounts of sulphuric and nitric acid constitutes the acid rain. The acid deposition is formed when large quantities of SO_2_ and NO_x_ are emitted to the atmosphere due to the combustion of fossil fuels [47,48]. These pollutants undergo chemical reaction in the atmosphere and form sulphuric (H_2_SO_4_) and nitric (HNO_3_) acids which come down as acid rain. There have been several studies on the constituents of the acid rain in different parts of the world and their impact on different types of plants [49,50,51,52,53,54]. Acid rain alters the chemical composition of the soil by leaching the base cations (such as Ca^2+^, Mg^2+^, K^+^ and Na^+^) with SO_4_^2−^ and NO_3_^−^ [55]. This affects not only the plants which thrive on the land but also the microbes which are present [55].

The specific aim of this paper is to explore the possibility of detecting these three pollutants, i.e., O_3_, NaCl and H_2_SO_4_, from the electrical response generated by the plants. For that purpose a set of experiments was conducted, using these three pollutants as external stimuli under laboratory conditions, on different species of plants, as presented in Reference [21] and their respective electrical responses were extracted and analyzed. 

In order to extract information about an underlying system from a raw time series, the entire duration of the series encompassing the trend may be considered for feature extraction (rather than windowing the time series, as applied in Reference [21,22]). These features, extracted from the entire duration of the time series, may then be used for classification. In this regard, coefficients from different curve fitting models can be looked at as potential features capturing the nature of the trend. The significance of these features as representative of underlying patterns in different time series can then be verified by the classification accuracies obtained. For this exploration, the same set of classification algorithms, i.e., LDA, QDA, Diaglinear, Diagquadratic and Mahalanobis distance were used as reported in Reference [21] in order to find out the best feature set-classifier combination. 

As a preliminary study, the curve fit coefficients from four different models, i.e., Polynomial, Gaussian, Fourier and Exponential were used, which captured the dynamics of the trend on the entire duration of the raw plant electrical signal response to three different stimuli, i.e., NaCl, H_2_SO_4_ and O_3_. Thereafter, these coefficients were treated as features and normalized similar to as explained in Reference [21]. However, no ranking of features was required in this case as the coefficients were taken together and not individually, which will be explained in detail in the following sections. Using the normalized coefficients as features, three sets of binary classification were carried out to evaluate the results. These results are shown in Section 5.

## 3. Methodology

### 3.1. Classification Algorithms

For work presented in this paper, five different classifiers—LDA, QDA, Diaglinear, Diagquadratic and Mahalanobis distance were used. 

#### 3.1.1. Discriminant Analysis

In cases where the class distinction is easily achievable, discriminant analysis classifiers such as Linear Discriminant Analysis (LDA) could be effective. Where such distinctions are not that straightforward, nonlinear classifiers such as kernel-based techniques like support vector machine (SVM) can be applied. A discriminant is a function which maps an output class label for an input variable. A discriminant function which is linear in the input variables is termed as linear discriminant function [56,57]. The aim of discriminant analysis is to reduce the dimensionality of the input feature space and project an n-dimensional input data (for n features) to a single dimensional space/line. However, not all projections will optimally separate the different classes. 

#### 3.1.2. LDA

LDA or linear discriminant analysis algorithm, takes in an input feature vector and it assigns it to one of the classes. The output of an LDA is given by (1) [56,57].

(1) $$y=\sum_{j=1}^{M}w_{j}{}^{T}f_{j}+b\;$$

Here, $w_{j}$ are the components of the weight vector $W_{a}$ (optimal projection) and $f_{j}$ are the components of the M dimensional feature vector F. b is the bias/offset which determines the location of the class separating hyperplane. The weight vector Wa determines the orientation of the hyperplane. Thus, a real number y is given as a linear combination of all the input features and their corresponding weights. This real number y, reflects the observation in reduced (single) dimension. LDA classifier performs well when the data is linearly separable. Where the data is not linearly separable, more complex decision boundaries may be required to separate the data [56,57]. For LDA, the covariance matrix is assumed to be identical for all classes and hence only one covariance matrix is estimated (pooled) for all classes [56,57]. 

#### 3.1.3. QDA

The linear discriminant function can be extended to include the products of pairs of components of the features (variables) and Equation (1) can be extended to (2).

(2) $$y=\sum_{j=1}^{M}w_{j}x_{j}+\sum_{j=1}^{M}\sum_{k=1}^{M}w_{jk}x_{j}x_{k}+b\;$$

Therefore, the additional $\frac{M\left(M+1\right)}{2}$ terms in the quadratic discriminant function in (2) allows more complex decision boundaries to be drawn between the classes [56,57]. For QDA, it is assumed that the covariance matrix is separate for each class and hence for each class, the covariance matrix is estimated separately [56,57].

#### 3.1.4. Diaglinear and Diagquadratic (Naïve Bayes) Classifier

Diaglinear and Diagquadratic classifiers, also known as Naïve Bayes method, is based on the assumption that the features (variables) are independent, thus ignoring any information sharing between the features. That is why the non-diagonal elements in the covariance matrix for the linear and the quadratic form (given by Equations (1) and (2)) are considered as zero and only the diagonal elements are taken into account [56,57]. 

#### 3.1.5. Mahalanobis Distance Classifier

Mahalanobis distance, instead of usual Euclidean distance, is a common measure of distance between two points (usually a point and the mean of the multivariate data) when variances are different in different directions. The Mahalanobis distance classifier is used to classify a point based on the least distance between the point and the class mean [56,57]. 

The Mahalanobis distance between a feature vector $x_{i}$ and the mean vector $m_{i}$, of a class i∈A,B is given in (3) and explained briefly below [56,57]:

(3) $$r_{i}=\sqrt{{\left(x_{i}-m_{i}\right)}^{T}\cdot \sum^{-1}\cdot \left(x_{i}-m_{i}\right)}\;$$

The estimate of the covariance matrix is denoted by ∑. If $r_{A}>r_{B}$, then the feature vector belongs to class B, otherwise it belongs to class A. In a 2D space (e.g., when two features are chosen), the region of constant Mahalanobis distance forms an ellipse. For higher than 2D space, ellipsoid or hyper ellipsoids are formed. The Mahalanobis distance of 1 unit corresponds to 1 standard deviation along all primary axes of variance (i.e., two primary axes when two features are chosen etc.). If ∑ happens to be identity matrix, then Mahalanobis distance turns out to be common Euclidean distance [56,57]. 

**Figure 1:**
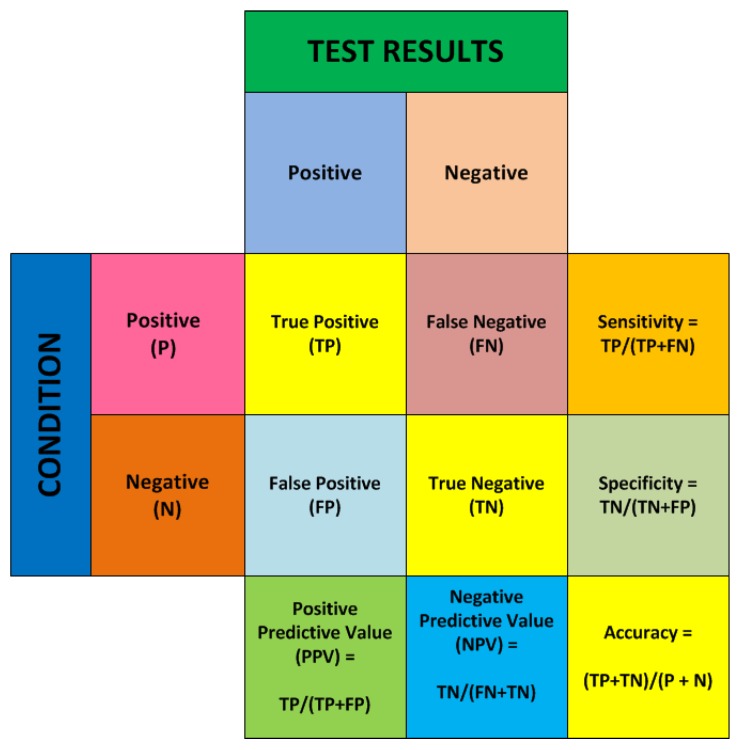
Confusion matrix—showing different measures of classification.

The parameters used during binary classification using curve fit coefficients is shown in Figure 1. In Binary Classification, when we were looking for a particular stimuli/class to be present—it was termed as *Positive*. So, when our classification algorithm correctly found the presence of a *Positive* out of a set of observations (i.e., the test dataset), it was called *True Positive* (TP)*.* When the algorithm detected a *Positive* out of the set, but in reality, it was not a *Positive*, this situation was called *False Positive.* Similarly, when the algorithm detected the presence of a *Negative* (i.e., the presence of the other class in a binary class setting) correctly, this was called *True Negative.* However, the detection of a *Negative*, where in reality it was a *Positive*, was called a *False Negative.* Figure 1 summarizes the conditions and the various measures which describe them. This figure is called the confusion matrix and is widely referred to for classification tasks [56,58].

The five measures—*Sensitivity*, *Specificity*, *PPV*, *NPV* and *Accuracy* were computed for all the binary classification scenarios in this exploration. Accuracy was taken into account to find out the classification success of any two different stimuli detected through the features extracted from the plant electrical signal response.

**Figure 2:**
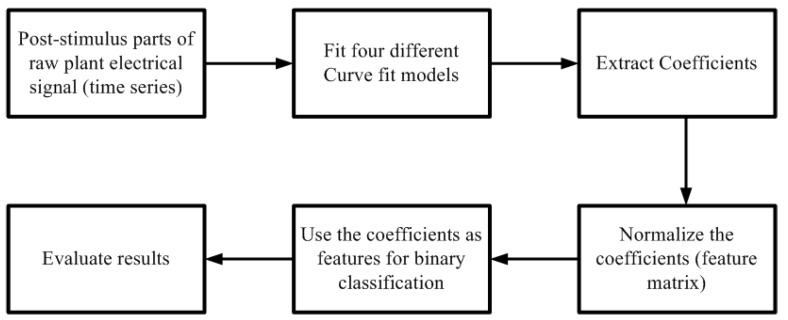
Classification using Curve fit coefficients.

The methodology used to extract the features from the raw signals and then use them for binary classification is shown in Figure 2. The curve fit coefficients were normalized similar to normalization of features as reported in Reference [21,22].

### 3.2. Curve Fitting Types

For this initial exploration to check if curve fit coefficients can be used as features for classifying stimuli affecting plant electrical signals, four curve fit types were chosen. These curve fit types were selected based on the visual observation of the plant electrical signal data available and a detailed reasoning is given under each curve fit model type described below.

**Figure 3:**
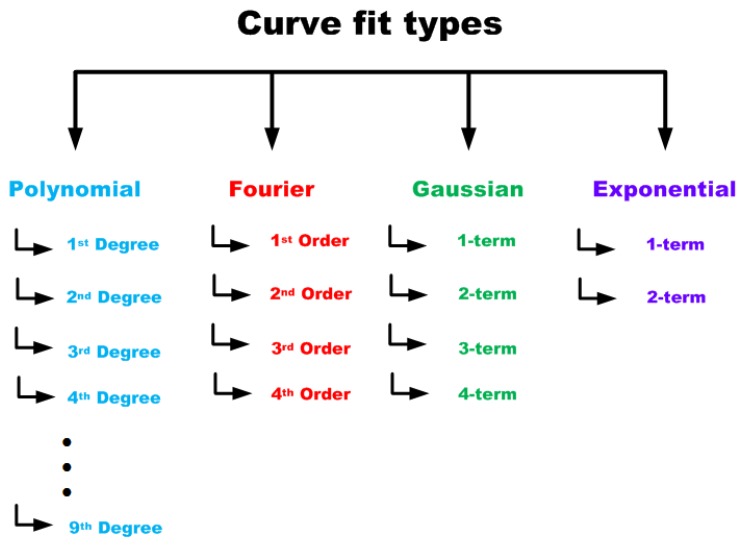
Four different curve fit types used to explore the coefficients as features for classification.

The four curve fit types chosen for the exploration are shown in Figure 3. As can be seen from this figure, for each of these curve fit types, a systematic variation in degree/order/terms were explored for extracting the coefficients which were then used for classification of the stimuli applied to the plants. The range of variation of the parameters are given in Table 1.

For this exploration, a script in MATLAB was written to fit the curves and extract the coefficients for the datasets directly rather than using the Curve Fitting Toolbox [59].

#### 3.2.1. Polynomial Curve Fit

Polynomial models for curve fits are given by (4), where n is the degree of the polynomial to be fitted to the time series. n+1 denotes the order of the polynomial which gives the number of coefficients to be fit. In our exploration, 1≤n≤9 keeps the number of coefficients (feature space) limited to 10, as the number of time series were low (total time series used for training were 411) as shown in Table 2.

(4) $$\overset{⌢}{y}=\sum_{i=1}^{n+1}P_{i}x^{n+1-i}\;$$

Polynomial fits are usually used for simple empirical models, especially for interpolation and extrapolation of data. However, in our case, it was used for characterizing the data using a global fit. One of the main disadvantages of polynomial fitting can be that the fit might be very good within a data range but can diverge outside this data range. However, since we used the coefficients as features across different time series and used them for classification, a good generic evaluation of the data fit was possible. 

It needs to be mentioned here that the MATLAB curve fit toolbox allows us to work around the scaling problem which arise when trying to fit data with high-degree polynomials by giving the option of *Centre and Scale* checkbox to alleviate the issues because of the great difference in scale between two inputs. However, to determine the classification results without using this feature, the given option was not exercised for this first exploration. As a future study, this may be used to see the difference in classification results. Also, the *Robust* linear least-squares fitting method was kept at default *Off.* Again, as a future study, various options such as LAR (Least Absolute Residual) or Bisquare (Bisquare weights method) could be used to check the difference in classification results. 

#### 3.2.2. Gaussian Curve Fit

The Gaussian model fits peaks of the data and is defined by (5)

(5) $$\overset{⌢}{y}=\sum_{i=1}^{n}a_{i}e^{\left[-{\left(\frac{x-b_{i}}{c_{i}}\right)}^{2}\right]}\;$$

Here, a is the amplitude, b is the centroid/location of the data, c is related to the peak width and n is the number of peaks to fit. In our case, 1≤n≤4 is used to keep the number of coefficients low due to low number of time series available for classification. 

#### 3.2.3. Fourier Curve Fit

The Fourier series is a sum of sine and cosine functions which is used to describe a periodic signal, as described by (6)

(6) $$\overset{⌢}{y}=a_{0}+\sum_{i=1}^{n}a_{i}\cos\left(i\omega x\right)+b_{i}sin\left(i\omega x\right)\;$$

Here, $a_{0}$ models the intercept (which is a constant) term in the data and is associated with i=0 cosine term. ω is the fundamental frequency of the signal and n is the number of terms (harmonics) used to fit the time-series. In our case, 1≤n≤4 is used to keep the number of coefficients low due to the low number of time series available for classification. 

#### 3.2.4. Exponential Model Curve Fit

The MATLAB curve fit capability provides one or two term exponential models which are given by (7)

(7) $$\left\{ \begin{array}{l} \overset{⌢}{y}=ae^{bx}\\ \overset{⌢}{y}=ae^{bx}+ce^{dx} \end{array}\right.\;$$

Exponentials are usually used to define time-series when the rate of change of quantity is proportional to the initial amount of the quantity. If the coefficient(s) b or d are negative, then it represents an exponential decay. 

## 4. Experimental Datasets

The datasets from experiments on Tomato, Cucumber and Cabbage plants using three different stimuli—NaCl, H_2_SO_4_ and O_3_ were obtained from Reference [60]. For each plant, three stainless steel needle electrodes—one at the base for reference, one in the middle and the other on top of the stem were used. The electrodes were 0.35 mm in diameter and 15 mm in length, similar to those used in EMG from Bionen S.A.S. and were inserted around 5–7 mm into the plant stem so that the sensitive active part of the electrodes (2 mm) were in contact with the plant cells. The electrodes were connected to the amplifier-Data Acquisition (DAQ) system as reported in References [21,22,61]. Plants were then enclosed in a plastic transparent box with proper openings to allow the presence of cables and inlet/outlet tubes and exposed to artificial light conditions (LED lights responding to plant’s photosynthetic needs, mimicking a day/night cycle of 12 h each). Each experiment was conducted in a dark room to avoid any external light interferences. The whole setup was then placed inside a Faraday cage to limit the effect of electromagnetic interference [1,2]. After the insertion of the electrodes into the plant, approximately 45 min of waiting period were allowed for the plant(s) to recover before starting the stimulations. Electrical signals acquired by the electrodes were provided as input to a 2-channel high impedance (1015 Ω) electrometer (DUO 773, WPI, Sarasota, FL, USA) while data recording was carried out through 4-Channel DAQ (LabTrax, WPI) and its dedicated software LabScribe (WPI) [62].

The sampling frequency was set to 10 samples per second for all the recordings (as it was deemed sufficient to capture the electrophysiological response of the plants appropriately). For the treatments with liquid, i.e., H_2_SO_4_ (5 mL, 0.05 M) or NaCl (5 or 10 mL of 3 M solution), a syringe was placed outside of the Faraday cage and connected to a silicone tube inserted into the plant soil. This was used to inject the solution. O_3_, produced by a commercial ozone generator (mod. STERIL, OZONIS, Cesano Maderno, Italy), [63] was injected into the box through a silicone tube (1-min spray every 2 h, 16 ppm), while a second outlet tube threw the O_3_ from the box to the chemical hood. The concentration of O_3_ inside the box was monitored using a suitable sensor [1,2]. 

There are many methods of extracting plant electrical signals. However, the two most common techniques employed to measure electrical signals in plants are extracellular and intracellular measurements [7]. Apart from these two, there are other techniques such as the aphid technique, patch-clamp recording technique, non-invasive microelectrode vibrating probe technique, and non-contact measurement using optical recording technique [7]. 

Intracellular measurement can be used to directly record the value of an individual cell plasma membrane potential, while extracellular measurement can be used to detect the spatiotemporal total of the depolarization–repolarization process in a large group of plant cells. Extracellular potential measurements are carried out on the surface of higher plants. One of the advantages of this technique is that it can be used to record potential differences over long periods (e.g., several days) because it does not involve any cell-altering electrolytes [7]. In higher plants, two types of extracellular measurements can be performed. These are measurements using *inserted metal electrodes*, and *surface recordings*. Measurement using metal electrodes inserted into the plants causes wounding reactions which can infiltrate or submerge reactions to external stimulus under study. Therefore the electrodes are required to have thin metal tips such as platinum (Pt) or silver/silver-chloride (Ag/AgCl)-wires of 0.4 to 1.0 mm in diameter [7]. When inserted into the shoot or leaf vein of the plants, the metal tips of the electrodes come in contact with tissues covering a larger group of cells and can support long term recording of electrical signal response [7]. Along with these techniques, researchers have also been looking at photochemical reflectance index (PRI) which has been increasingly used in precision agriculture [17] to monitor the plant’s photosynthetic process which can be affected by environmental stressors such as salt/drought/temperature stress etc. A refinement of such techniques will enable remote monitoring of the plant’s conditions against a variety of environmental stressors and indirectly turn the plants into environmental biosensors. 

For the exploration, the number of time series (note: Only the post-stimulus parts were used) is given in Table 2. Few of these experimental datasets were new and not used in previous studies [21,22]. Data from each channel was considered as a separate time series (two channels per plant were used per experiment) and care was taken to use new plants for every separate experiment. That is, eight plants for NaCl (two channels per plant, single application of stimuli), O_3_ (20 different plants for two channels per plant, 5/9/10 or 11 stimuli), H_2_SO_4_ (two channels per plant, 13 plants, single/double application of stimuli) were used. Also as explained in previous studies [21,22], in the case of O_3_ as stimulus—each experiment consisted of multiple exposure of the stimulus. Each duration between stimuli were considered as a separate time series, as sufficient time (approx. 60 min) was allowed between exposures.

The entire duration of the post-stimulus part for each time series was used to fit the four different curve fitting models. 

**Figure 4:**
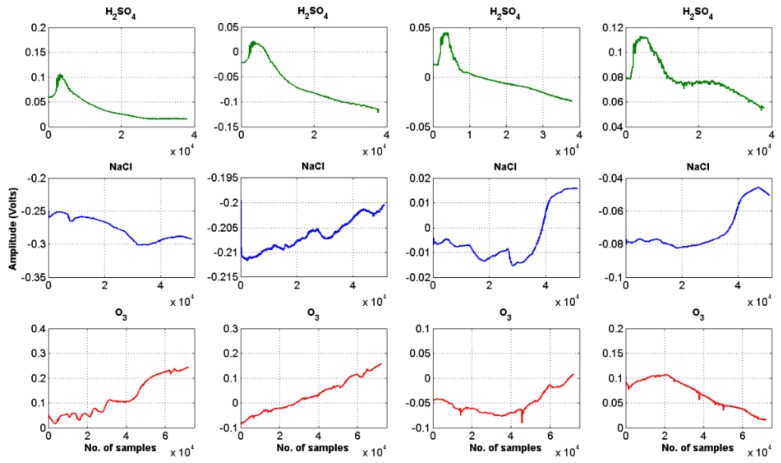
Raw plant electrical signal response after application of three types of stimuli.

Four sets of raw data, from the three stimuli are shown in Figure 4 as an example. 

## 5. Results and Discussion

**Figure 5:**
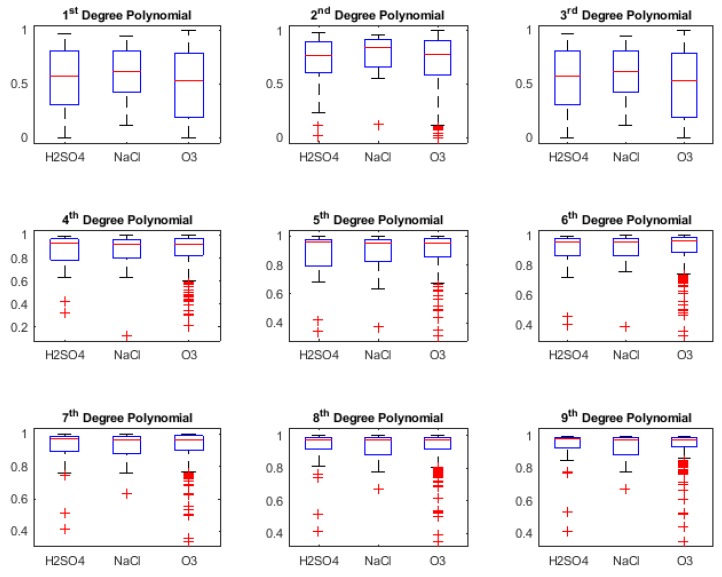
R-squared values for Polynomial curve fitting.

**Figure 6:**
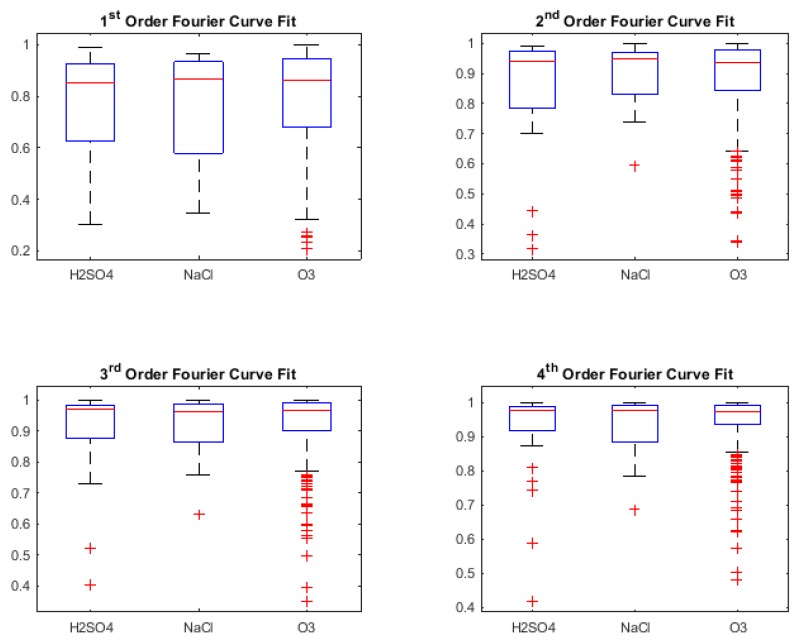
R-squared values for Fourier curve fitting.

**Figure 7:**
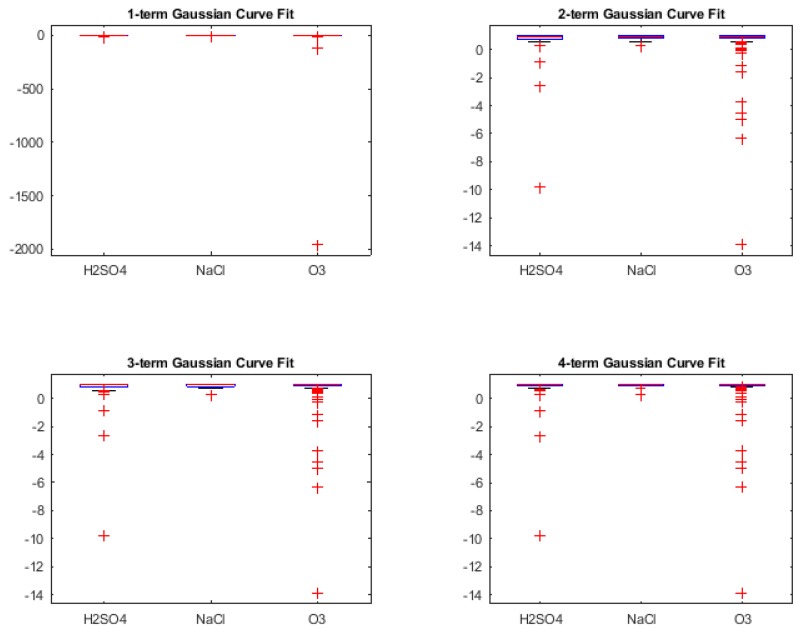
R-squared values for Gaussian curve fitting.

**Figure 8:**
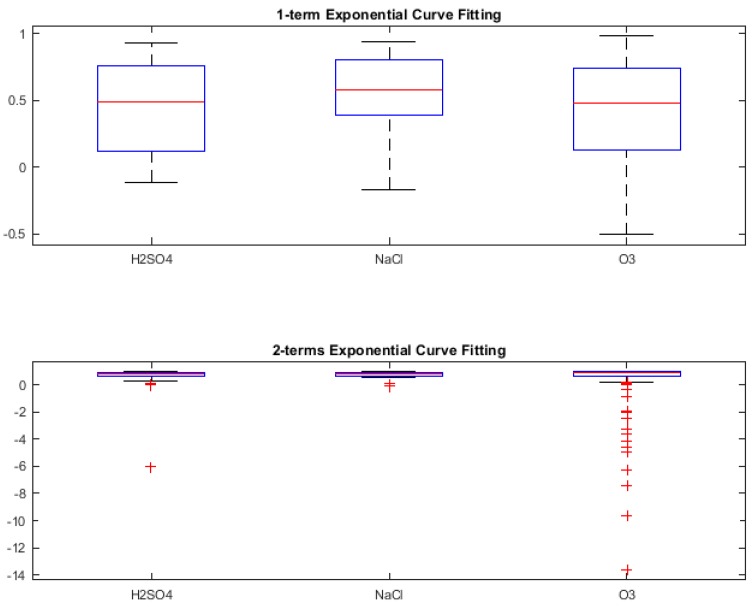
R-squared values for Exponential curve fitting.

In this section, we present the classification results obtained during retrospective study (i.e., using Leave One Out Cross-Validation (LOOCV) to check the classifier capability during training phase) [56,57] for three different binary stimuli combinations. However, it was also necessary to look at the *Goodness of fit* of the various curve fit types, in order to understand what kind of result for classification can be expected. These are shown using box-plots of the *R-squared* values of the fit for the types of curves as shown in Figure 5, Figure 6, Figure 7 and Figure 8. R-squared is used as a statistical measure to find out how close the actual data is to the fitted regression line. By definition [64],

(8) $$R-squared=\frac{Explained\_Variation}{Total\_Variation}\;$$

and its value always ranges between 0 and 1 (i.e., 100%). A value of 0 indicates that the chosen model cannot explain any of the variability of the data centered on its mean, whereas a value of 1 indicates that the chosen model explains all the variability within the data. As a consequence, a higher R-squared value translates to a better fit for the model to the data [64]. In our case, the R-squared values for each curve fitting type for the three stimuli have been shown using Box-Plots to get a good overview of the goodness of fit. This way, we can relate to any classification results to the accuracy of the curve fit coefficients in capturing the morphology of the plant electrical signal response data which occurred due to the application of the particular stimuli. 

Figure 5 shows the R-squared values for Polynomial curve fit, ranging from 1st to 9th degree, for three different stimuli which were applied to the plants. 

From Figure 5, it can be clearly seen that for Polynomial degree 1 and 3, the median of the R-squared values lies around 0.5. For degree 2, although the median is higher than 0.5, the range of lowest to highest values (shown by the whiskers) are large for H_2_SO_4_ and O_3_. This improves for Polynomial 4th degree onwards; however, we see lots of outliers for O_3_ in particular. 

Looking at the R-squared values for Fourier curve fits in Figure 6, the median can be seen above 0.8 with the interquartile range getting narrower with the increment in the order, denoting consistent fit. However, the outliers are more visible as the order is increased. 

For Gaussian and Exponential curve fits, the R-squared values as seen from Figure 7 and Figure 8 respectively, lie very close to 0 with increment in number of terms. The negative R-squared values indicate the chosen model fits worse than a horizontal line, is the wrong model to fit the data and does not follow the trend which is present in the raw signal. Hence, the coefficients from Gaussian and Exponential were not proceeded with, for using as features for classification. 

**Figure 9:**
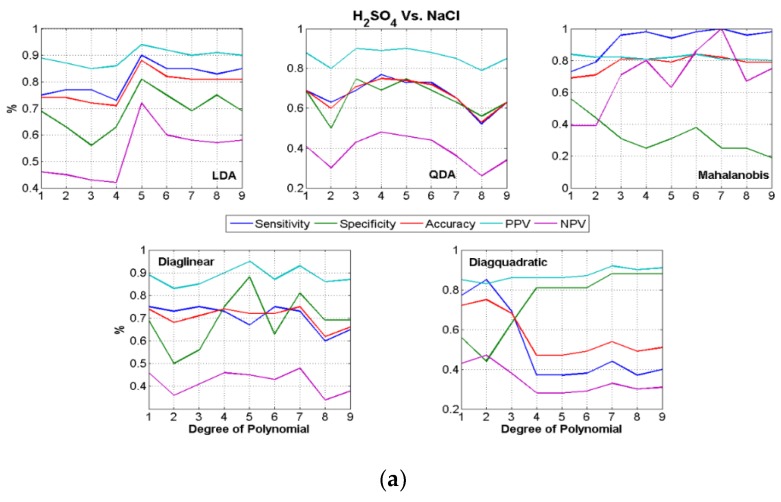

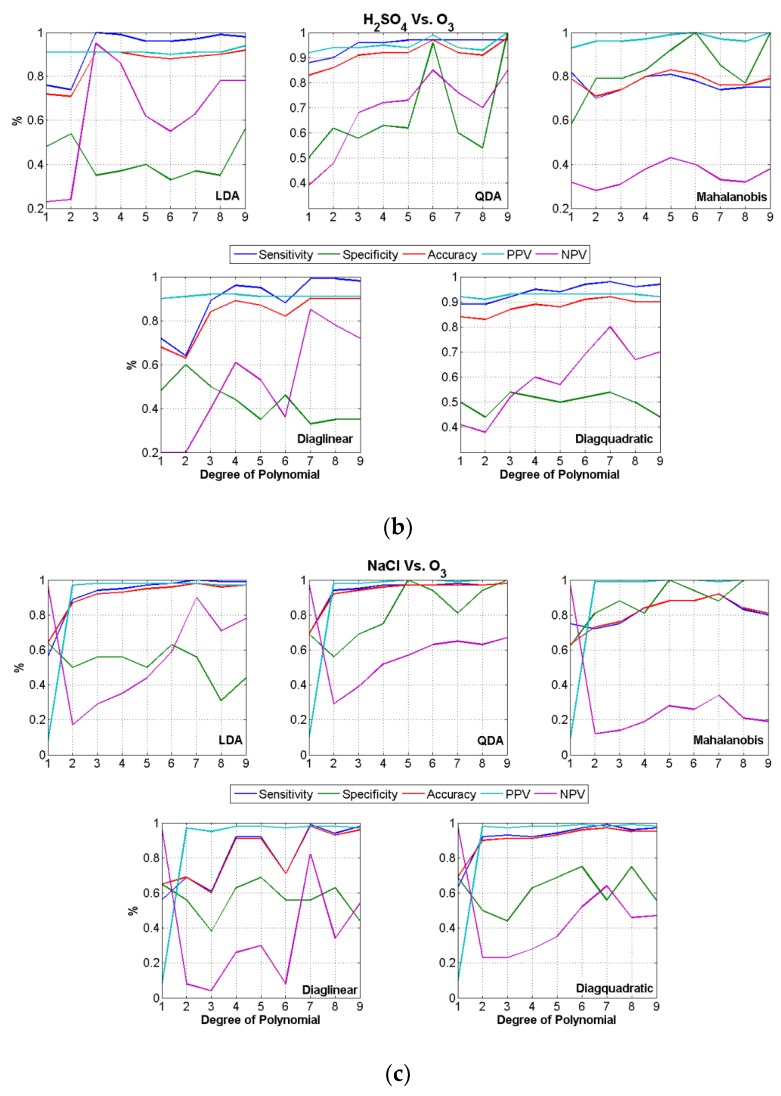
Binary classification results using Polynomial Curve Fit Coefficients.

**Figure 10:**
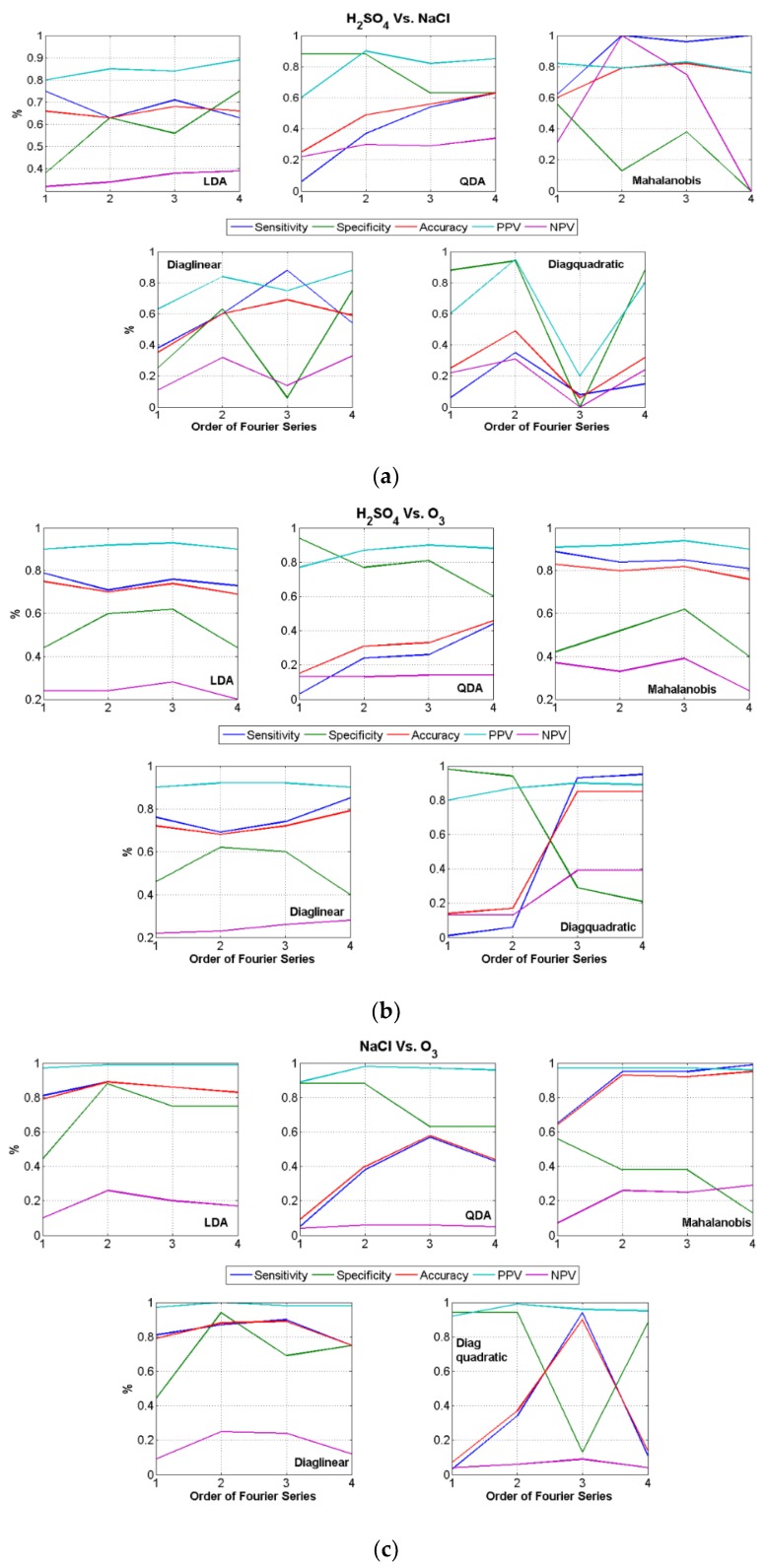
Binary classification results using Fourier Curve Fit Coefficients.

The binary classification results (three combinations using three stimuli) are carried out, using each of the curve fit coefficients as features for classification. The results need to be evaluated by looking at sensitivity, specificity and accuracy together (defined previously), to get a feel for the performance of the classifiers in detecting both classes (i.e., stimuli) rather than just accuracy which can be artificially high if one of the two classes are misclassified [56]. Beginning with Polynomial curve fit coefficients which are shown in Figure 9a–c, where each sub-figure shows the variation in degree of the curve fit type and for each classifier type (five variants used as explained earlier). The figures also show the plots of the accuracy, sensitivity, specificity, PPV and NPV as explained in Figure 1. The values on the y-axis of Figure 9 and Figure 10 need to be multiplied by 100 to get the actual value in percentage. That is, a value of 0.1 denotes 10% whereas a value of 0.9 denotes 90%. 

From Figure 9a–c, it is observed that the best classification results are obtained by using Polynomial coefficients of degree 5 or above for all the five parameters measured. These are consistent for all the five variants of classifiers used, although LDA and QDA outperform other classifiers. 

Similarly, Figure 10a–c shows the classification results using the coefficients of the Fourier curve fit using different terms. Again, for all the five variants of the classifiers used, all five parameters such as sensitivity, specificity etc. are shown. These parameters show a higher value for 3rd or 4th terms, although the results are not as consistent as obtained for Polynomial curve fits. That is, the accuracy is found to be high in few cases but either sensitivity or specificity were low. This signifies that using just Fourier coefficients, classification of all the three stimuli were difficult. 

The sensitivity, specificity and accuracy has been found to be poor for all the three binary stimuli combinations when using coefficients from Gaussian and Exponential curve fits. These were as expected from the R-squared values for Gaussian and Exponential curve fits as presented earlier. Hence, the classification results obtained using the coefficients from Gaussian and Exponential are not reported in this paper.

From the figures above, we can see that out of all the various results obtained for three different types of binary classification combinations (retrospective study), using coefficients from different curve fit types, the best results obtained were obtained only while using Polynomial coefficients and these results are summarized in Table 3.

As a next step, in order to test the effectiveness of the classification obtained so far on training data, four timeseries belonging to each stimulus were kept separate from the training phase (retrospective study). Based on the classification results given in Table 3, a *One versus One* (OVO) decision tree was designed to the test the held-out data (which was not used for training the classifiers). Using this decision tree, the result which was obtained is given in Table 4. These four time-series were used for testing (prospective study) the classification system identified (i.e., feature-classifier combination).

**Figure 11:**
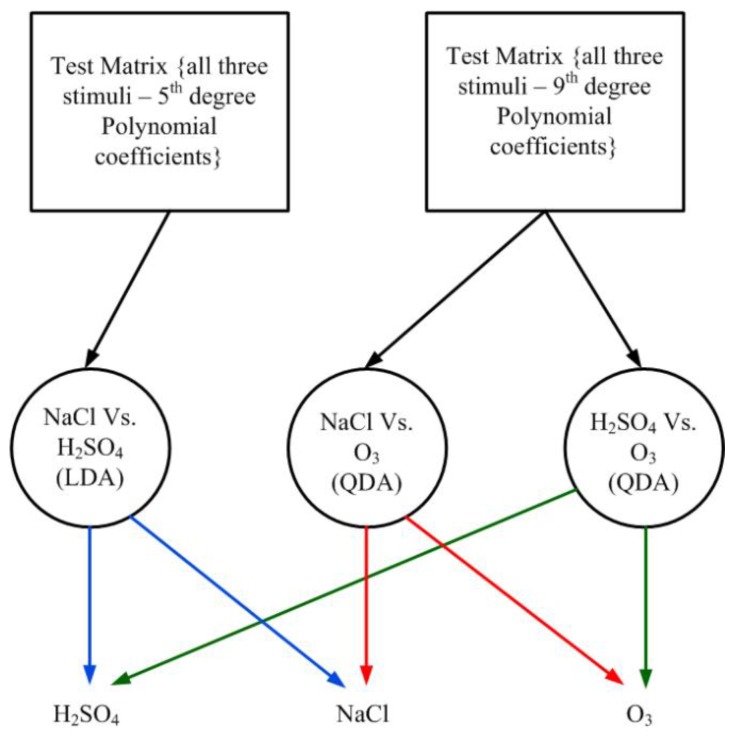
Prospective test method using One Versus One classification decision tree.

The test matrix was designed by including the coefficients required for each binary stimulus combination, as given in Table 3, for all the three stimuli. This is shown in Figure 11. This way, it was easy to send a train of the coefficients of the required degree for testing which stimuli it belonged to. The final classification of the test dataset was done using a custom designed OVO Decision tree [56]. The prospective study results are given in Table 4.

As can be seen, for totally unseen dataset, only two out of four held out data were correctly classified in case of H_2_SO_4_ and NaCl, whereas all four held out datasets were correctly classified for O_3_. This is possibly due to the availability of more O_3_ data (343 time-series) for the classifiers to be trained on than H_2_SO_4_ (52 time-series) and NaCl (16 time-series). The O_3_ data included as much variation in the distribution as possible for the new data to be correctly classified. Whereas in case of H_2_SO_4_ and NaCl, insufficient data did not introduce as much training required for the classifiers as possible.

However, classification using polynomial curve fit coefficients produced good retrospective results and it also produced a good prospective result for O_3_ as a stimulus. The underlying trend in the data were better captured using the Polynomial model rather than Fourier, Gaussian or Exponential. Although the Goodness of fit using the Fourier curve fit was closer to Polynomial, the classification results were not as consistent. The prospective results were obtained using polynomial coefficients of degrees 5 and 9. However, if a single degree of polynomial-classifier was to be picked as a generic feature-classifier combination, then it has to be coefficients of polynomial degree 9 and QDA classifier as identified from Figure 9a–c. These results using such a combination during training phase is given in Table 5.

There was no change in prospective results using this above feature-classifier configuration. 

## 6. Summary

In this paper, classification using Curve fit coefficients have been explored. The classification was carried out exactly as explained previously in Reference [21], i.e., using LOOCV on binary stimuli combinations. Coefficients from 5th (one binary stimuli combination) and 9th (two binary stimuli combination) degree Polynomial curve fit as features were found to be producing the best classification results. These results were then used to design an OVO decision tree to test the few held out data, out of which—H_2_SO_4_ and NaCl were classified only 2/4 times. However, using the same decision tree, O_3_ were detected 4/4 times. This can be attributed to lesser training data for the former two stimuli. As a generic feature-classifier combination, Polynomial degree 9 and QDA was found to be suitable. However, this combination provided comparatively lower accuracy for H_2_SO_4_ vs. NaCl as can be seen from Table 5. Moreover, there was no change in prospective study results using this generic feature-classifier combination. 

The conclusion is that coefficients of the Polynomial curve fitting models can be used for the classification of stimuli as they capture the entire trend of the raw plant electrical signal response. This raw signal trend adds more information to the signal than the stochastic part alone, explored in References [22,65]. Although only 12 time series were available to test the decision tree, the comparison is not exact with those results obtained using the segmentation method presented in References [21,22] due to the larger number of training and testing samples available. The exploration presented in this paper provides another way to extract features from raw plant electrical signals, which resulted in 98% classification accuracy in the retrospective study. These results are much better than the binary classification results in References [21,22]. This was expected due to the longer duration of the time series used to extract features which provides more information about the applied stimuli. However, the segmentation method does provide the insight that any segment from the entire plant electrical signal response provides enough information to the classifier about the stimuli affecting the plant.

Comparing the classification accuracy (with high sensitivity and specificity values) with those reported in Reference [22], the results obtained using curve fit coefficients were found to be much better. Due to the limited datasets available for training, the prospective study resulted in only 50% classification accuracy for NaCl and H_2_SO_4_. Since more time series were available for training, a classification accuracy as high as 100% for O_3_ was achieved.

## Figures and Tables

**Table 1:** Curve fit types and parameters.

Fit Type	Degree/No. of Terms	Fit Options (Robust, Algorithm, etc.)
Polynomial	1≤n≤9	MATLAB Default
Gaussian	1≤n≤4	MATLAB Default
Fourier	1≤n≤4	MATLAB Default
Exponential	1≤n≤2	MATLAB Default

**Table 2:** Number of time series used for each stimulus.

Stimulus	Number of Time Series
NaCl	16
H_2_SO_4_	52
O_3_	343

**Table 3:** Best Binary Classification results (retrospective study) using Curve fit coefficients.

Binary Stimuli Combination	Fit Type	Degree	Classifier	Classification Results		
				Sensitivity	Specificity	Accuracy
H_2_SO_4_ vs. NaCl	Polynomial	5th	LDA	90%	81%	88%
H_2_SO_4_ vs. O_3_	Polynomial	9th	QDA	97%	100%	98%
NaCl vs. O_3_	Polynomial	9th	QDA	98%	100%	98%

**Table 4:** Results from prospective study using separate held out data.

Stimuli	Total Held Out Time Series Used for Prospective Testing	Number of Correctly Classified Time Series
H_2_SO_4_	4	2
NaCl	4	2
O_3_	4	4

**Table 5:** Classification results using 5th Degree Polynomial and QDA Classifier.

Binary Stimuli Combination	Fit Type	Degree	Classifier	Classification Results		
				Sensitivity	Specificity	Accuracy
H_2_SO_4_ vs. NaCl	Polynomial	9	QDA	63%	63%	63%
H_2_SO_4_ vs. O_3_	Polynomial	9	QDA	97%	100%	98%
NaCl vs. O_3_	Polynomial	9	QDA	98%	100%	98%
